# Explainable Machine Learning for Predicting Complicated Appendicitis and Expected Hospital Length of Stay in Children: An Exploratory Single-Center Study

**DOI:** 10.3390/medicina62081438

**Published:** 2026-07-24

**Authors:** Ahmad Turki, Enas Raml, Jury Emad Aboulola

**Affiliations:** 1 Electrical and Computer Engineering Department, Faculty of Engineering, King Abdulaziz University, Jeddah 21589, Saudi Arabia; 2 Center of Excellence in Intelligent Engineering Systems (CEIES), King Abdulaziz University, Jeddah 21589, Saudi Arabia; 3 Pediatric Surgery Department, King Abdullah Medical Complex and the Specialized Maternity and Children’s Hospital, Jeddah 23816, Saudi Arabia; eraml@moh.gov.sa; 4 Department of Medicine, Batterjee Medical College, Jeddah 21442, Saudi Arabia; 24110499@bmc.edu.sa

**Keywords:** pediatric appendicitis, appendicolith, perforated appendicitis, acute abdomen, surgical outcomes, feature engineering, machine learning, predictive modeling, supervised learning, clinical decision support systems

## Abstract

*Background and Objectives*: Pediatric appendicitis remains a common surgical emergency, but early risk stratification of complicated appendicitis and expected hospital length of stay (LOS) remains challenging. This study developed and internally evaluated an explainable machine learning framework for pediatric appendicitis using routinely available clinical, laboratory, and radiological variables. *Materials and Methods*: This retrospective single-cohort model-development study included 152 pediatric patients with acute appendicitis treated at King Abdulaziz University Hospital, Jeddah, Saudi Arabia, between January 2019 and December 2024. Two prediction tasks were evaluated: complicated appendicitis classification and expected LOS regression. Prediction was performed after initial clinical assessment, first laboratory testing, and initial diagnostic imaging, but before surgery or definitive conservative treatment. Operative findings, histopathological findings, postoperative variables, treatment-response variables, actual LOS-related variables, and final disease-severity labels were excluded as predictors. Model performance was evaluated using repeated nested cross-validation, bootstrap 95% confidence intervals, calibration analysis, benchmark comparisons, LOS sensitivity analyses, and explainability analysis using feature importance and SHAP values. *Results*: Complicated appendicitis was present in 57 patients (37.5%), and median LOS was 3 days (IQR: 2–6). For complicated appendicitis prediction, the repeated nested cross-validation framework achieved an AUC of 0.788 (95% CI: 0.710–0.856), accuracy of 0.750 (95% CI: 0.684–0.816), sensitivity of 0.684 (95% CI: 0.559–0.796), specificity of 0.789 (95% CI: 0.705–0.868), and Brier score of 0.183 (95% CI: 0.158–0.210). The framework showed comparable performance to a parsimonious logistic regression model (AUC: 0.807). For expected LOS prediction, the regression framework achieved an MAE of 2.150 days (95% CI: 1.632–2.846), MdAE of 1.305 days (95% CI: 1.004–1.493), RMSE of 4.329 days (95% CI: 2.290–6.297), and R^2^ of 0.292 (95% CI: 0.213–0.481). LOS prediction showed lower MAE, MdAE, and RMSE than median and mean LOS null baseline models. Explainability analysis identified symptom duration, lymphocyte percentage, vomiting, temperature, and age as important predictors of complicated appendicitis, while radiological perforation, symptom duration, radiological collection, CRP, WBC count, NLR, lymphocyte percentage, and appendicolith by radiology contributed to expected LOS prediction. *Conclusions*: Explainable machine learning methods showed potential for internally validated prediction of complicated appendicitis and expected LOS using post-assessment pre-treatment data. However, this was a retrospective, single-cohort development study with a modest sample size and no external validation cohort. The findings should therefore be interpreted as exploratory and should not be considered evidence of clinical generalizability or readiness for implementation. Larger prospective multicenter studies with external validation, prediction-interval estimation, and clinical utility assessment are required before clinical use.

## 1. Introduction

Acute appendicitis is one of the most common pediatric surgical emergencies and remains a frequent cause of emergency department visits, hospital admission, antibiotic therapy, and urgent abdominal surgery worldwide [1,2]. Although the disease is common, diagnosis and risk stratification remain challenging in children because clinical presentation varies by age, symptom duration, communication ability, appendix position, and stage of inflammation [3,4]. Younger children may present with nonspecific symptoms, atypical pain localization, or delayed recognition, which can increase the risk of perforation and other complicated forms of disease [3,4]. Delayed diagnosis and progression of inflammation are clinically important because complicated appendicitis is associated with abscess formation, intra-abdominal collection, peritonitis, longer hospitalization, greater antibiotic exposure, and increased healthcare utilization [5,6]. In Saudi pediatric practice, diagnostic uncertainty remains clinically relevant; a recent local study reported complicated appendicitis in 30.9% of pediatric appendicitis cases and a normal appendix rate of 13.4%, highlighting the need for improved risk stratification in local clinical settings [7].

Current assessment of suspected pediatric appendicitis relies on the integration of clinical history, physical examination, laboratory markers, and imaging. Commonly used clinical and laboratory indicators include right lower quadrant pain, vomiting, anorexia, fever, leukocytosis, neutrophilia, neutrophil-to-lymphocyte ratio (NLR), and C-reactive protein (CRP) [8]. However, no single symptom, sign, or inflammatory marker is sufficiently reliable in isolation, particularly when children present early or with atypical symptoms [8]. Imaging, especially ultrasound, is recommended as a first-line modality in many pediatric settings because it can identify appendiceal enlargement, non-compressibility, appendicolith, periappendiceal inflammatory change, free fluid, abscess, or collection while avoiding ionizing radiation [9,10]. Nevertheless, ultrasound is operator-dependent and may be nondiagnostic in some patients because of body habitus, bowel gas, appendix location, early disease, or advanced inflammatory changes [9,10]. Therefore, pediatric appendicitis decision-making often requires synthesis of multiple heterogeneous data sources.

Clinical scoring tools were developed to support this synthesis and improve bedside risk stratification. Samuel introduced the Pediatric Appendicitis Score (PAS), a simple point-based diagnostic score combining symptoms, physical signs, fever, leukocytosis, and neutrophilia to estimate the probability of appendicitis in children [11]. Kharbanda et al. later developed the Pediatric Appendicitis Risk Calculator (pARC), which provides a continuous probability estimate using demographic, clinical, examination, and laboratory variables [12]. Cotton et al. externally validated pARC in community emergency departments and reported that it accurately stratified appendicitis risk in children aged five years and older [13]. The RIFT study further emphasized the importance of prospective multicenter validation of appendicitis risk prediction tools in children presenting with right iliac fossa pain [14]. These tools are clinically useful because they are interpretable, practical, and available during initial assessment. However, PAS and pARC were primarily designed to estimate the probability of appendicitis, not to predict downstream outcomes such as complicated appendicitis or expected hospital length of stay (LOS). Therefore, in the present study, an incompletely reconstructed PAS was used only as a limited exploratory contextual comparison rather than as a validated clinical benchmark. Machine learning (ML) provides a complementary approach because it can integrate heterogeneous clinical, laboratory, and radiological variables and model nonlinear relationships that may not be fully captured by traditional scoring systems or conventional regression models [15]. Recent systematic reviews have shown increasing interest in artificial intelligence (AI) and ML for appendicitis diagnosis, severity assessment, imaging interpretation, treatment planning, and postoperative prognosis [16,17,18]. Issaiy et al. reviewed AI models for diagnostic and prognostic appendicitis tasks and emphasized that many models show promising performance but require stronger validation before clinical implementation [16]. Chekmeyan and Liu specifically reviewed AI for pediatric appendicitis diagnosis and concluded that AI may improve diagnostic accuracy, but it remains an evolving technology requiring standardized study design, reporting, and real-world evaluation [17]. Maleš et al. reviewed AI applications across the appendicitis care pathway, including triage, diagnosis, severity prediction, treatment selection, and postoperative management [18].

Original pediatric appendicitis ML studies have also demonstrated the potential of data-driven prediction tools. Marcinkevics et al. used ML to predict diagnosis, management strategy, and severity in pediatric appendicitis using clinical and laboratory data [19]. In later work, Marcinkevičs et al. developed interpretable and intervenable ultrasound-based ML models, showing that human-understandable imaging concepts can support prediction of diagnosis, management, and severity in suspected pediatric appendicitis [20]. Chadaga et al. developed an interpretable ML framework for pediatric appendicitis detection and used explainability methods to identify influential predictors [21]. Byun et al. used CT, laboratory, and clinical features to predict complicated appendicitis in children, supporting the value of multimodal preoperative data for severity prediction [22]. Reismann et al. applied artificial intelligence methods to routinely collect clinical parameters to diagnose pediatric appendicitis and distinguish complicated from uncomplicated disease [23]. Maleš et al. showed that explainable ML may help reduce negative appendectomies in pediatric patients with high clinical probability of appendicitis while also supporting discrimination of complicated disease [24]. More recent pediatric studies by Erman et al. and Manohar et al. further demonstrate the growing role of ML for preoperative severity and management prediction, particularly when clinical markers and explainability techniques are incorporated [25,26].

Several gaps remain in pediatric appendicitis prediction research. Most machine learning studies focus on diagnosis, severity, perforation, or treatment selection, while fewer evaluate hospital length of stay (LOS) as a continuous outcome. Early LOS prediction is challenging because it is influenced by both baseline disease characteristics and later clinical events; therefore, prediction timing and predictor availability must be clearly defined [27,28]. Complex algorithms should also be compared with simpler, interpretable models such as parsimonious logistic regression, particularly in modest single-center datasets where limited sample size and outcome events increase the risk of overfitting [29,30,31,32,33]. TRIPOD and TRIPOD+AI further emphasize transparent reporting, calibration, uncertainty assessment, and external validation [34,35]. Finally, explainability methods such as SHAP may help confirm that predictions are driven by clinically plausible variables rather than post-treatment or cohort-specific patterns [36,37].

To address these gaps, the present exploratory study internally evaluated explainable machine learning methods for predicting complicated appendicitis and expected hospital length of stay (LOS) using routinely available clinical, laboratory, and radiological variables from a Saudi pediatric cohort. A single prespecified prediction time point was defined after the initial clinical assessment, first laboratory investigations, and initial diagnostic imaging, but before surgery or definitive conservative treatment; therefore, the models should be interpreted as early post-assessment, pre-treatment tools rather than admission-triage models. To contextualize performance and reduce overinterpretation, the complicated appendicitis framework was compared with a modified Pediatric Appendicitis Score and a parsimonious conventional logistic regression model, while LOS prediction was compared with cohort median and mean null baselines. Model performance was evaluated using repeated nested cross-validation, calibration metrics, bootstrap confidence intervals, LOS sensitivity analyses, and explainability methods.

## 2. Materials and Methods

### 2.1. Study Design and Population

This study used a retrospective observational model-development design to internally evaluate an explainable machine learning framework for pediatric appendicitis. The analysis focused on two clinically relevant pre-treatment prediction tasks: prediction of complicated appendicitis and prediction of expected hospital length of stay (LOS).

To reduce predictor-timing bias and improve clinical interpretability, a single prespecified prediction time point was defined for both models. Prediction was performed after completion of the initial emergency clinical assessment, first laboratory investigations, and initial diagnostic imaging, but before surgery or definitive conservative treatment. Therefore, only variables available at this pre-treatment time point were eligible for model development.

Candidate predictors included demographic variables, presenting symptoms, symptom duration, initial vital signs, first laboratory results, and pre-treatment radiological findings. Operative findings, histopathological findings, postoperative variables, treatment-response variables, final disease-severity labels, actual LOS-related variables, and hospitalization-course variables were excluded from model development. In addition, variables directly used to define complicated appendicitis were excluded from the complicated appendicitis prediction model to reduce target leakage and circular prediction.

The final analytical cohort included 152 pediatric patients diagnosed with acute appendicitis. Patients were managed either surgically or conservatively according to clinical judgment and institutional practice.

### 2.2. Inclusion and Exclusion Criteria

Consecutive electronic health records were screened for pediatric patients diagnosed with acute appendicitis at King Abdulaziz University Hospital, Jeddah, Saudi Arabia, between January 2019 and December 2024. A total of 200 pediatric patient records were assessed for eligibility. Patients were included if they were 4–14 years of age, had a confirmed or clinically managed diagnosis of acute appendicitis, had available hospital length of stay (LOS) data, and had sufficient pre-treatment clinical, laboratory, and radiological data for analysis. Both surgically managed and conservatively managed patients were eligible for inclusion.

Of the 200 screened records, 48 were excluded: 12 for missing key outcome data, 22 for incomplete essential medical records, 7 duplicate encounters, and 7 for underlying chronic conditions expected to independently affect hospital LOS. These chronic conditions included malignancy, immunodeficiency, or major systemic illness (Table 1).

**Figure 1:**
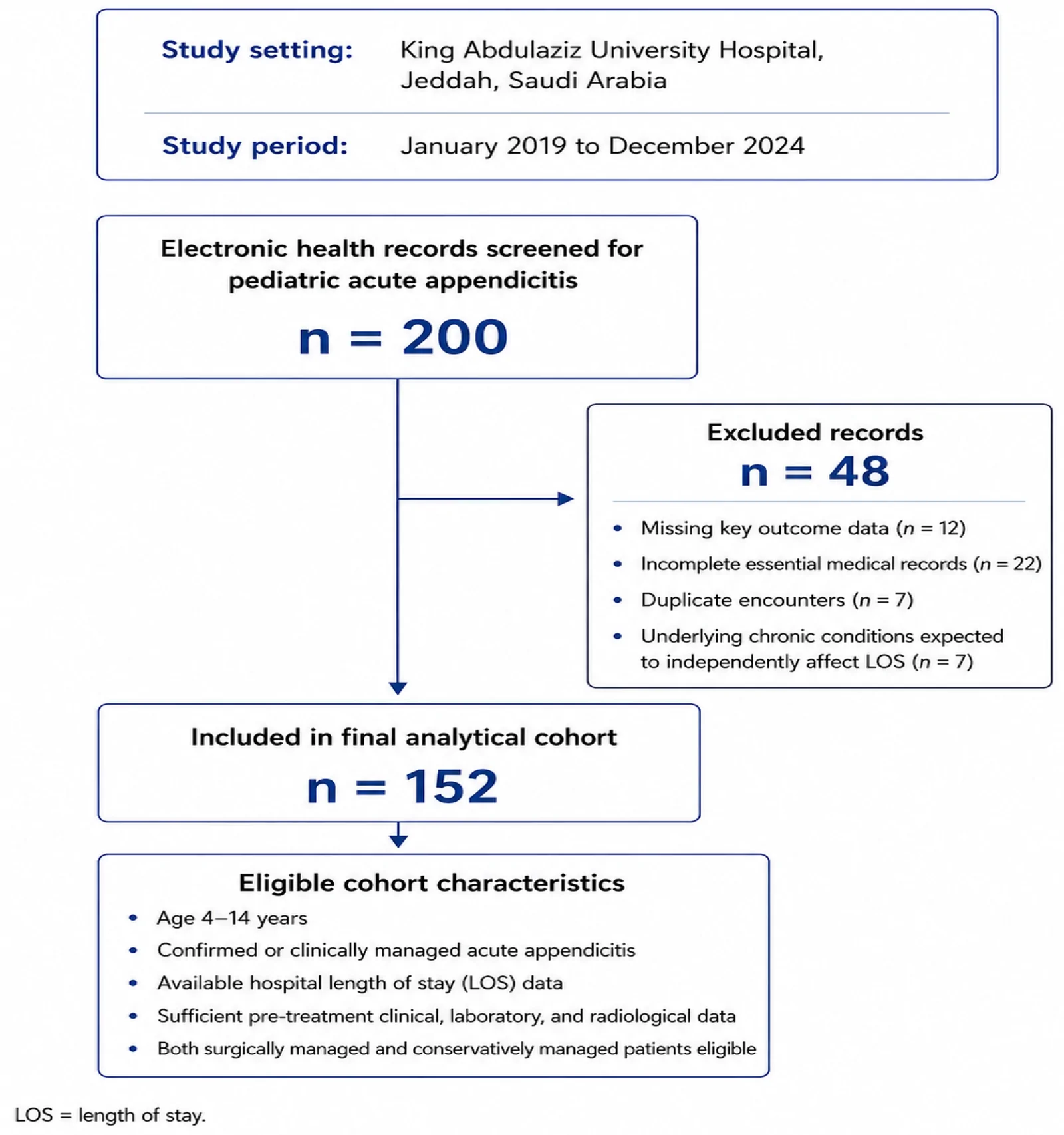
Patient flow diagram for cohort selection.

After exclusions, 152 patients were included in the final analytical cohort. The patient selection process is summarized in Figure 1.

Electronic health records were screened for pediatric acute appendicitis at King Abdulaziz University Hospital between January 2019 and December 2024. Of 200 screened records, 48 were excluded, and 152 patients were included in the final analytical cohort. LOS: length of stay.

### 2.3. Ethical Approval

This study was approved by the Bioethics Committee of the Center of Excellence in Intelligent Engineering Systems (CEIES), King Abdulaziz University, under approval number 28-CEIES-Bioeth-2025. The approved project involved a retrospective dataset of pediatric appendicitis cases, including structured clinical, laboratory, and radiological variables for machine learning analysis. The approval was granted according to the ethical guidelines adopted by the National Bioethical Committee of Saudi Arabia and was valid from 20 June 2025 to 20 June 2027.

The study was conducted using anonymized retrospective clinical data. Data collection and patient-consent procedures followed the forms and requirements approved by the CEIES Bioethics Committee. All patient data were anonymized before analysis, and no personally identifiable information was included in the final analytical dataset.

### 2.4. Data Collection

Clinical data were extracted from electronic health records at King Abdulaziz University Hospital, King Abdulaziz University, Jeddah, Saudi Arabia, for pediatric patients diagnosed with acute appendicitis between January 2019 and December 2024. The extracted data were organized into a structured dataset for statistical analysis, machine learning model development, and explainability analysis.

Demographic variables included age and sex. Clinical presentation variables included abdominal pain, vomiting, anorexia, fever, diarrhea, nausea, constipation, dysuria, cough, and scrotal pain. Symptom duration before presentation, fever documented in the emergency room, and body temperature at presentation were also recorded.

Laboratory variables included white blood cell count, neutrophil percentage, lymphocyte percentage, neutrophil-to-lymphocyte ratio (NLR), and C-reactive protein (CRP). Laboratory predictors were restricted to the first available laboratory results obtained during the initial clinical assessment.

Pre-treatment radiological variables included appendicolith, perforation, free fluid, fat stranding, abscess, phlegmon, and intra-abdominal or pelvic collection when documented on the initial diagnostic imaging report.

All patients underwent abdominal ultrasound as the initial diagnostic imaging modality. Computed tomography (CT) was not routinely performed and was reserved for patients in whom an intra-abdominal or pelvic collection was identified or suspected on ultrasound, to confirm and further characterize the finding. Accordingly, 124 patients (81.6%) underwent ultrasound alone, whereas 28 patients (18.4%) underwent ultrasound followed by CT. Radiological findings were retrospectively extracted from the original pre-treatment ultrasound and, when applicable, CT reports using a structured data-collection form. No independent retrospective rereview of the images was performed. When both modalities were available, findings from both pre-treatment reports were considered, with the CT report used to confirm and characterize collection-related abnormalities.

Operative and histopathological variables, including appendiceal inflammation, gangrene, perforation, localized or generalized pus, abscess, intra-abdominal collection, transmural necrosis, periappendicitis, fibrosis, and pathological abscess formation, were extracted for outcome classification and descriptive comparison only.

The primary outcomes were complicated appendicitis and expected hospital length of stay (LOS). Complicated appendicitis was analyzed as a binary classification outcome, while LOS, measured in days, was analyzed as a continuous regression outcome.

### 2.5. Data Cleaning and Preprocessing

The dataset underwent structured cleaning and preprocessing before statistical analysis and machine learning model development. Records were reviewed for duplicate encounters, incomplete entries, inconsistent formatting, spelling variations, non-standard units, and clinically implausible values. Duplicate records representing the same clinical encounter were removed.

Numerical variables stored as text were converted to numeric format. Age was recorded in years, while symptom duration and hospital length of stay (LOS) were recorded in days. Continuous variables, including age, symptom duration, body temperature, WBC count, neutrophil percentage, lymphocyte percentage, NLR, CRP, and LOS, were checked for formatting errors, inconsistent units, and clinically implausible values. When unclear values were identified, the original electronic health record was reviewed and corrected when possible.

Numerical predictor standardization was performed only when required by the model. Standardization was fitted using training data only within the cross-validation procedure and then applied to the corresponding validation or test fold. In the repeated nested cross-validation analysis, preprocessing steps were included within the modeling pipeline so that scaling and model fitting were performed separately within each training fold of the inner and outer cross-validation loops.

### 2.6. Outcome Definitions

The study focused on two primary prediction outcomes: complicated appendicitis and hospital length of stay (LOS). Complicated appendicitis was analyzed as a binary classification outcome, while hospital LOS was analyzed as a continuous regression outcome. The regression model was designed to estimate expected LOS from pre-treatment predictors.

Hospital LOS was measured in days and defined as the number of calendar days from hospital admission to hospital discharge. LOS was used only as the continuous regression outcome and was not used as an input predictor in any machine learning model.

Complicated appendicitis was defined using predefined radiological, operative, and histopathological severity criteria. Patients were classified as having complicated appendicitis if they had at least one of the following findings: appendiceal perforation; gangrenous appendicitis; periappendiceal or intra-abdominal abscess; intra-abdominal or pelvic collection; phlegmon; generalized purulent peritonitis; or histopathological evidence of transmural necrosis, perforation, or abscess formation. Patients with any of these findings were coded as 1, representing complicated appendicitis. Patients without these findings were coded as 0, representing uncomplicated appendicitis.

For surgically managed patients, complicated appendicitis classification was based on operative findings and histopathological reports when available. For conservatively managed patients without operative or histopathological assessment, classification was based on predefined severity findings documented on the initial diagnostic imaging report, including perforation, abscess, phlegmon, or intra-abdominal/pelvic collection.

Prolonged hospitalization and composite adverse clinical outcomes were not modeled as separate machine learning prediction tasks. This decision was made to maintain consistency of predictor timing, avoid circular prediction, and reduce interpretation problems related to heterogeneous post-treatment outcomes. Outcome rates were calculated as shown in Equation (1):

(1) $$\mathrm{Outcome}\;\mathrm{Rate}(\%)=\frac{\mathrm{Number}\;\mathrm{of}\;\mathrm{patients}\;\mathrm{with}\;\mathrm{the}\;\mathrm{outcome}}{\mathrm{Total}\;\mathrm{number}\;\mathrm{of}\;\mathrm{patients}}\times 100$$

### 2.7. Statistical Analysis

Descriptive statistics were used to summarize patient demographics, clinical presentation, laboratory findings, pre-treatment radiological findings, treatment approach, and outcomes. Continuous variables were summarized using mean and standard deviation or median and interquartile range, depending on the distribution of the data. Categorical variables were summarized as frequencies and percentages.

Patients were stratified into uncomplicated and complicated appendicitis groups according to the predefined outcome definition. Demographic, clinical, laboratory, pre-treatment radiological, and outcome variables were compared between groups. Continuous variables were compared using the independent-samples t-test for normally distributed variables or the Mann–Whitney U test for non-normally distributed variables. Categorical variables were compared using the chi-square test or Fisher’s exact test when expected cell counts were small.

Associations between pre-treatment clinical, laboratory, and non-target-defining radiological predictors and complicated appendicitis were assessed using univariate analysis. Odds ratios and 95% confidence intervals were calculated for clinically relevant binary predictors of complicated appendicitis. Univariate analyses were restricted to non-outcome-defining pre-treatment predictors. LOS was analyzed as a continuous outcome; therefore, odds ratios were not calculated for LOS.

Machine learning model development, repeated nested cross-validation, performance metrics, calibration analysis, and bootstrap confidence intervals are described separately in Section 2.8 and Section 2.9. A *p*-value less than 0.05 was considered statistically significant.

### 2.8. Machine Learning Model Development

Machine learning models were developed for two prediction tasks: complicated appendicitis as a binary classification outcome and expected hospital length of stay (LOS) as a continuous regression outcome. Predictor eligibility followed the prespecified pre-treatment prediction time point described in Section 2.1. The final predictors included in each model are presented in Table 2.

Expected LOS prediction was expressed as shown in Equation (2):

(2) $${\hat{LOS}}_{i}=f(X_{i})$$

where ${\hat{LOS}}_{i}$ is the predicted hospital LOS for patient i, $X_{i}$ represents the input-feature vector for patient i, and f represents the trained machine learning prediction function.

For complicated appendicitis classification, logistic regression, support vector machine (SVM), random forest, gradient boosting, and extreme gradient boosting (XGBoost) were evaluated. Hyperparameters were selected by grid search within the inner cross-validation loop. Logistic regression tuning included the regularization parameter; SVM tuning included the regularization parameter and kernel coefficient; random forest tuning included the number of trees, maximum tree depth, and minimum samples per leaf; gradient boosting tuning included the number of estimators, learning rate, and maximum tree depth; and XGBoost tuning included the number of estimators, maximum tree depth, learning rate, subsampling, and column subsampling.

For expected LOS prediction, ridge regression, random forest regression, and gradient boosting regression were evaluated. Ridge regression tuning included the regularization strength. Random forest regression tuning included the number of trees, maximum tree depth, feature-sampling strategy, and minimum samples per leaf. Gradient boosting regression tuning included the number of estimators, learning rate, maximum tree depth, minimum samples per leaf, and loss function. A fixed random state of 42 was used for applicable models to improve reproducibility.

An exploratory contextual comparison was performed using an incompletely reconstructed Pediatric Appendicitis Score. A complete PAS could not be calculated because several physical-examination components were not consistently documented in the retrospective records. Right-lower-quadrant tenderness was coded as present for all included patients, migration of pain was coded as absent when unavailable, and cough, percussion, or hopping tenderness was assigned a score of zero when undocumented. Because these assumptions do not reproduce a prospectively measured PAS, the reconstructed score was used only as a limited exploratory comparison and not as a validated clinical benchmark. The parsimonious logistic regression benchmark included symptom duration, fever documented in the emergency department, WBC count, NLR, CRP, and appendicolith on initial imaging. This benchmark was used to determine whether the broader machine learning framework provided additional predictive value beyond a simpler and clinically interpretable model.

For expected LOS prediction, two null baseline models were evaluated. These models predicted either the cohort median LOS or the cohort mean LOS for every patient and were used to determine whether the machine learning regression models improved prediction beyond simple cohort-level estimates.

Because LOS was right-skewed and included one extreme hospitalization of 42 days, two sensitivity analyses were performed. First, the repeated nested cross-validation analysis was repeated after excluding the 42-day observation. Second, a log-transformed model was evaluated using log(LOS+1) as the regression outcome. Predictions from the log-transformed model were back-transformed to days before calculating performance metrics.

Model performance was evaluated using repeated nested cross-validation. For complicated appendicitis classification, the outer loop consisted of stratified five-fold cross-validation repeated 10 times, resulting in 50 held-out test folds. Within each outer training set, stratified five-fold inner cross-validation was used for model and hyperparameter selection based on the area under the receiver operating characteristic curve. The outer test folds were used only for performance estimation and were not involved in preprocessing, model selection, or hyperparameter tuning.

For expected LOS regression, the outer loop consisted of five-fold cross-validation repeated 10 times, also resulting in 50 held-out test folds. Within each outer training set, five-fold inner cross-validation was used for model and hyperparameter selection based on mean absolute error. The held-out outer folds were used exclusively for performance estimation.

The final analytical cohort contained no missing values for the predictors or outcomes used in model development; therefore, imputation was not required. Standardization was fitted using only the training data within each cross-validation fold and then applied to the corresponding validation or test fold. Standardization was used for logistic regression, SVM, and ridge regression, whereas tree-based models were trained using unstandardized predictors. All preprocessing steps were incorporated into the cross-validation pipeline to prevent information from validation or test patients from entering model development.

Class imbalance in the complicated appendicitis task was addressed using balanced class weights for logistic regression, SVM, and random forest. For gradient boosting and XGBoost, balanced sample weights were calculated from the outcome distribution within each training fold and supplied during model fitting.

Each patient received multiple out-of-fold predictions across the repeated outer loops. Patient-level predicted probabilities for complicated appendicitis and predicted LOS values were averaged across repeats before final performance evaluation. Because of the modest cohort size, the analyses were considered exploratory and internally validated.

All predictors were required to be available at the prespecified pre-treatment prediction time point. Radiological perforation and intra-abdominal or pelvic collection were excluded from the complicated appendicitis model because they contributed directly to the outcome definition. Operative, histopathological, treatment-related, postoperative, hospitalization-course, and outcome variables were excluded from both prediction models.

### 2.9. Model Performance Evaluation

Expected hospital length of stay (LOS) prediction was evaluated using mean absolute error (MAE), median absolute error (MdAE), root mean squared error (RMSE), and coefficient of determination ($R^{2}$). MdAE was included as a robust measure less influenced by extreme LOS values. These metrics were calculated as follows:

(3) $$MAE=\frac{1}{N}\sum_{i=1}^{N}|LOS_{i}-{\hat{LOS}}_{i}|$$

(4) $$MdAE=median\left(|LOS_{i}-{\hat{LOS}}_{i}|\right)\;\;\;\;\;\;\;\;\;\;\;\;\;\;\;\;\;\;$$

(5) $$RMSE=\sqrt{\frac{1}{N}\sum_{i=1}^{N}(LOS_{i}-{\hat{LOS}}_{i}{)}^{2}}$$

(6) $$R^{2}=1-\frac{\sum_{i=1}^{N}(LOS_{i}-{\hat{LOS}}_{i}{)}^{2}}{\sum_{i=1}^{N}(LOS_{i}-\bar{LOS}{)}^{2}}$$

where $R^{2}$ is the coefficient of determination, $LOS_{i}$ is the observed LOS for patient i, ${\hat{LOS}}_{i}$ is the predicted LOS for patient i, $\bar{LOS}$ is the mean observed LOS, and N is the number of patients.

Complicated appendicitis classification was evaluated using accuracy, sensitivity, specificity, precision, F1-score, area under the receiver operating characteristic curve (AUC), and Brier score. A prespecified probability threshold of 0.50 was used for threshold-dependent metrics. AUC was used to assess discrimination across probability thresholds and was calculated from continuous predicted probabilities.

The Brier score was used to assess the accuracy of predicted probabilities, as shown in Equation (7):

(7) $$Brier\;score=\frac{1}{N}\sum_{i=1}^{N}(y_{i}-{\hat{p}}_{i}{)}^{2}$$

where $y_{i}$ is the observed binary outcome and ${\hat{p}}_{i}$ is the predicted probability of complicated appendicitis. Calibration was assessed using the calibration intercept, calibration slope, and calibration plot.

The machine learning classification framework was compared with the modified Pediatric Appendicitis Score and the parsimonious logistic regression benchmark. The modified PAS was evaluated using AUC and threshold-dependent classification metrics; the Brier score was not calculated because PAS is a point-based score rather than a calibrated probability model. The parsimonious logistic regression model was evaluated using the same classification metrics as the machine learning models.

LOS models were compared with null models that predicted either the cohort median or cohort mean LOS for all patients. These baselines were evaluated using MAE, MdAE, RMSE, and $R^{2}$.

Sensitivity analyses evaluated LOS performance after excluding the 42-day hospitalization and after modeling log(LOS+1), with predictions back-transformed to days before metric calculation.

All performance metrics were calculated from patient-level out-of-fold predictions generated during repeated nested cross-validation and averaged across repeats. Ninety-five percent confidence intervals were estimated using patient-level bootstrap resampling with 2000 samples.

Formal prediction intervals and decision-curve analysis were not performed. Therefore, clinical usefulness was interpreted using discrimination, calibration, benchmark comparisons, and prediction-error metrics. Because no external validation cohort was available, the results represent internal validation only and require confirmation in an independent cohort before clinical implementation.

### 2.10. Explainable AI Analysis

Explainable AI analyses were performed to identify the predictors contributing most strongly to model outputs. For complicated appendicitis, logistic regression was used because it was the most frequently selected classification model across the outer cross-validation folds and provided transparent coefficient-based interpretation. Predictor importance was assessed using standardized coefficients, while linear SHAP values quantified the direction and magnitude of individual predictor contributions.

For expected hospital length of stay (LOS), gradient boosting regression was used because it was the most frequently selected regression model. Global feature importance and tree-based SHAP values were calculated to assess predictor contributions to estimated LOS.

After performance evaluation, both models were refitted using the complete analytical dataset solely for explainability analysis and not for performance estimation. Logistic regression predictors were standardized before refitting.

The SHAP explanation represents each model prediction as the sum of a baseline prediction and the contribution of each input feature, as shown in Equation (8):

(8) $$f(x)=\phi_{0}+\sum_{j=1}^{M}\phi_{j}$$

where f(x) is the model output for a given patient, $\phi_{0}$ is the baseline model output, M is the number of input predictors, and $\phi_{j}$ is the contribution of predictor j to the model output. Coefficients, feature importance, and SHAP values were interpreted as predictive associations rather than causal effects.

All statistical analyses, machine learning model development, repeated nested cross-validation, bootstrap confidence interval estimation, calibration analysis, benchmark comparisons, sensitivity analyses, explainability analyses, and figure generation were performed using Python 3 (Python Software Foundation, Wilmington, DE, USA).

## 3. Results

### 3.1. Patient Demographics and Baseline Characteristics

A total of 152 pediatric patients diagnosed with acute appendicitis were included in the analysis. The mean age was 9.64 ± 2.22 years, with a median age of 10 years and a range of 4–14 years. Male patients represented 65.8% of the cohort. The median symptom duration was 1 day, and the median hospital length of stay (LOS) was 3 days.

### 3.2. Comparison Between Uncomplicated and Complicated Appendicitis

Patients were classified as having uncomplicated or complicated appendicitis according to predefined radiological, operative, and histopathological severity criteria. Complicated appendicitis was defined by the presence of at least one of the following findings: appendiceal perforation, gangrenous appendicitis, periappendiceal or intra-abdominal abscess, intra-abdominal or pelvic collection, phlegmon, generalized purulent peritonitis, or histopathological evidence of transmural necrosis, perforation, or abscess formation. For conservatively managed patients without operative or histopathological assessment, classification was based on predefined severity findings documented on the initial diagnostic imaging report, including perforation, abscess, phlegmon, or intra-abdominal/pelvic collection.

Based on this definition, 95 patients (62.5%) had uncomplicated appendicitis and 57 patients (37.5%) had complicated appendicitis.

Patients with complicated appendicitis had significantly longer symptom duration, higher WBC count, higher neutrophil percentage, lower lymphocyte percentage, higher NLR, and longer LOS than patients with uncomplicated appendicitis. Fever documented in the emergency room was also significantly more common in the complicated group. Appendicolith by radiology was more frequent among patients with complicated appendicitis but did not reach statistical significance.

Severity-defining variables, including perforation, radiological collection, and pathological abscess, are shown for descriptive comparison only. These variables were not used as predictors in the complicated appendicitis machine learning model because they directly contributed to the outcome definition.

### 3.3. Hospital Length of Stay Outcomes

**Figure 2:**
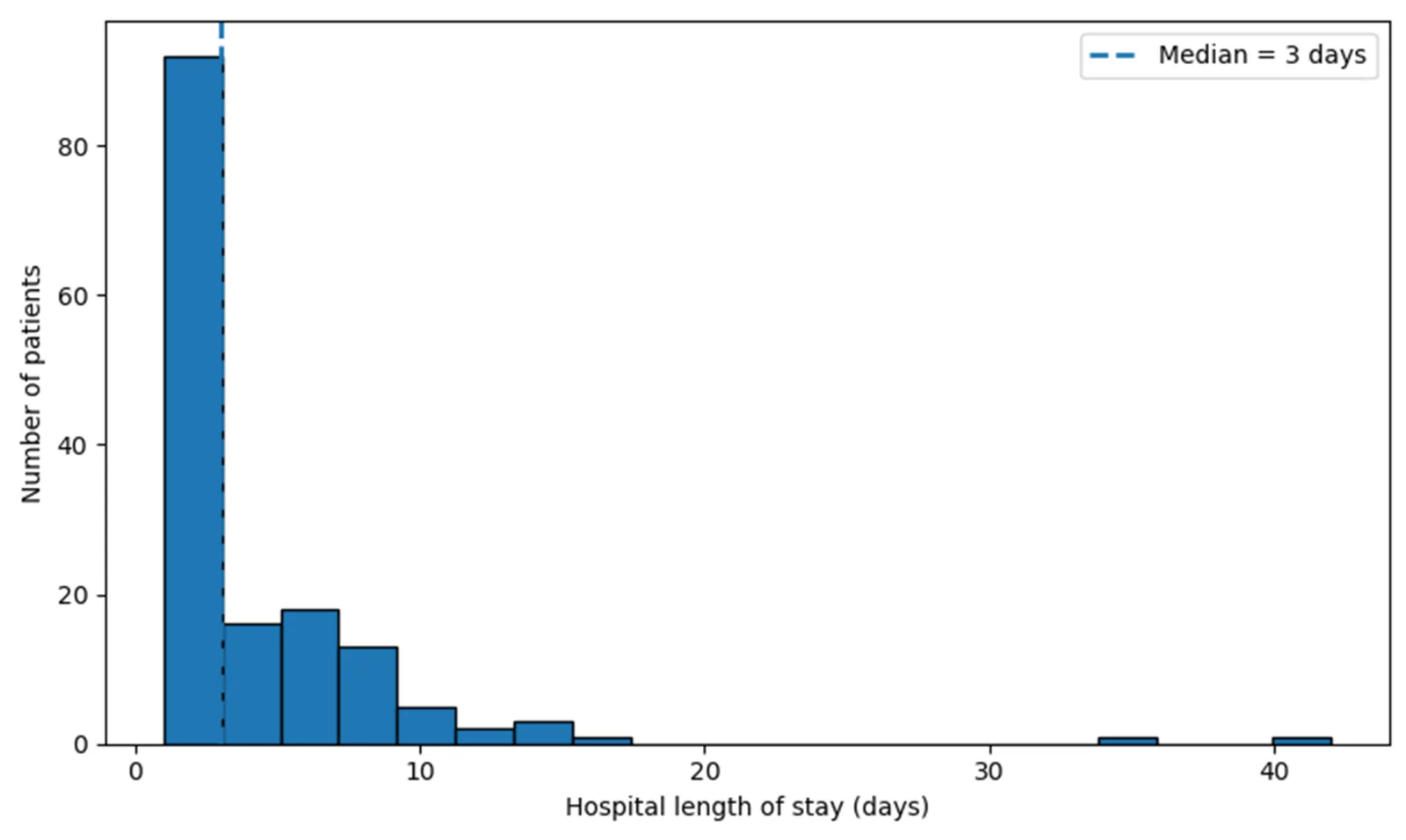
Distribution of hospital length of stay.

Hospital length of stay (LOS) was analyzed as a continuous outcome. The median hospital LOS was 3 days (IQR: 2–6 days), with a mean of 4.45 ± 5.16 days and a range of 1–42 days. The distribution was right-skewed, with a median LOS of 3 days and a maximum observed LOS of 42 days (Figure 2), with most patients discharged within the first few days and a small number of patients experiencing substantially longer hospitalization.

**Figure 3:**
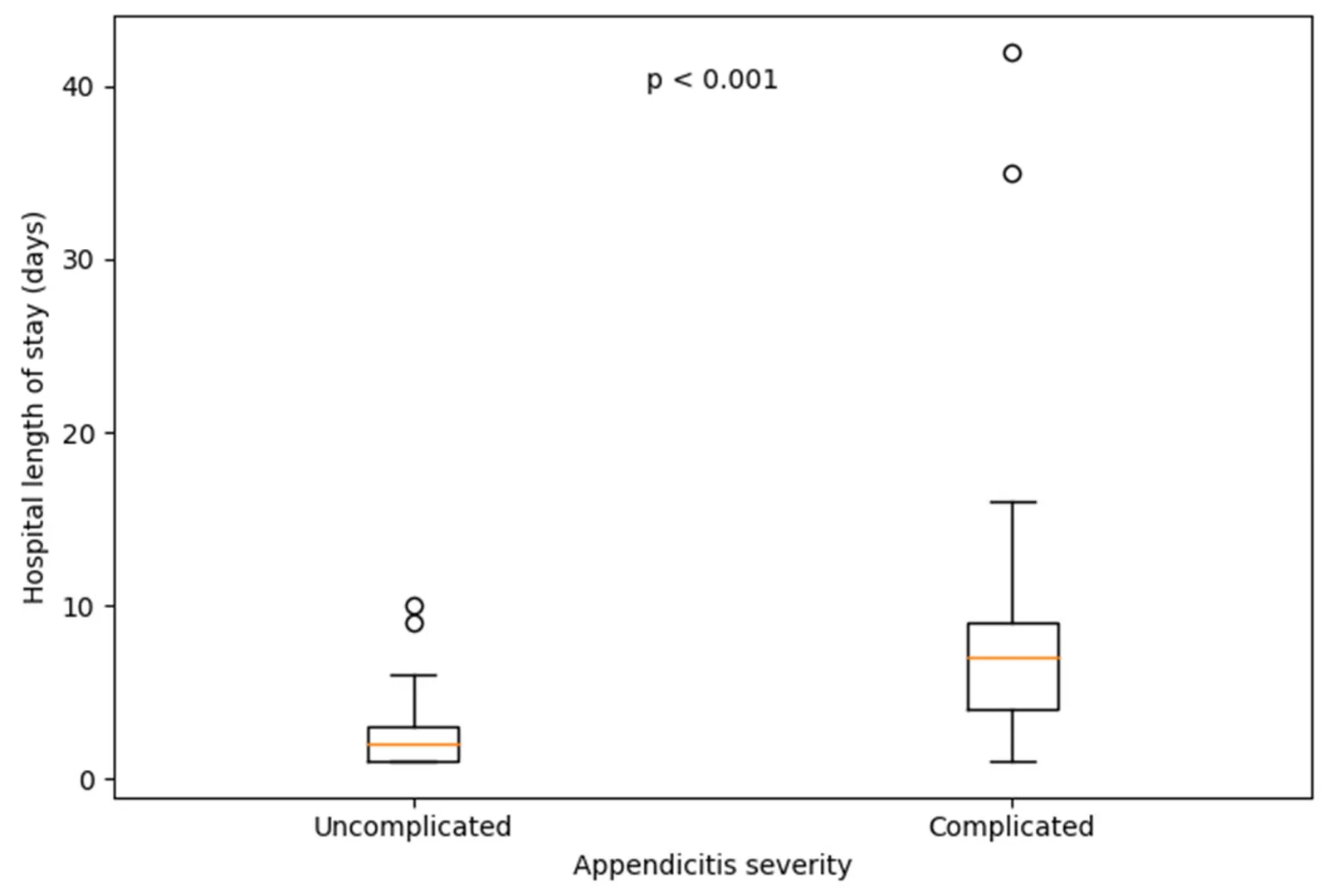
Hospital length of stay according to appendicitis severity. The orange horizontal lines represent the median hospital length of stay, the boxes rep-resent the interquartile range, the whiskers extend to values within 1.5 times the inter-quartile range, and the circles indicate outliers. Hospital length of stay was significantly longer in patients with complicated appendicitis

Patients with complicated appendicitis had significantly longer LOS than patients with uncomplicated appendicitis. The median LOS was 7 days (IQR: 4–9 days) in the complicated appendicitis group compared with 2 days (IQR: 1–3 days) in the uncomplicated appendicitis group (*p* < 0.001; Table 3 and Figure 3). These findings support the clinical association between appendicitis severity and hospitalization duration (Table 4).

Although prolonged hospitalization, defined as LOS > 7 days, occurred in 26 patients (17.1%), it was not modeled as a separate machine learning prediction task. LOS was instead analyzed as a continuous regression outcome to avoid duplicating hospitalization-related endpoints and to maintain consistency with the final two-task prediction framework.

Figure 3 shows a boxplot comparing LOS between uncomplicated and complicated appendicitis groups. Patients with complicated appendicitis had significantly longer LOS than patients with uncomplicated appendicitis.

### 3.4. Predictors of Complicated Appendicitis

Univariate analysis was performed to identify pre-treatment clinical, laboratory, and non-target-defining radiological variables associated with complicated appendicitis. Continuous variables were dichotomized using cohort-based thresholds for descriptive odds ratio analysis: symptom duration > 1 day, WBC count > 14.0 × 10^9^/L, NLR > 8.2, and CRP > 0.67 mg/dL. These thresholds were used only for univariate analysis and were not used to define the machine learning predictor set. Appendicolith was included as a non-target-defining radiological variable because it was not used to define complicated appendicitis.

Symptom duration > 1 day was strongly associated with complicated appendicitis, with an odds ratio of 5.35. Fever documented in the emergency room, WBC count > 14.0 × 10^9^/L, and NLR > 8.2 were also significantly associated with complicated appendicitis. CRP > 0.67 mg/dL and appendicolith by radiology were not statistically significant.

Variables directly used to define complicated appendicitis, including perforation, radiological collection, abscess, phlegmon, gangrene, transmural necrosis, and pathological abscess, were not included in this predictor analysis because they represent outcome-defining severity features rather than non-target-defining pre-treatment predictors.

### 3.5. Machine Learning Performance for Complicated Appendicitis

Machine learning models were developed to predict complicated appendicitis using variables available at the prespecified pre-treatment prediction time point. Candidate predictors included age, sex, presenting symptoms, symptom duration, initial vital signs, first laboratory results, and non-target-defining pre-treatment radiological findings. Variables directly used to define complicated appendicitis, including perforation, intra-abdominal or pelvic collection, abscess, phlegmon, gangrenous appendicitis, transmural necrosis, pathological abscess, and operative or pathological severity findings, were excluded from the predictor set to reduce target leakage and circular prediction.

Repeated nested cross-validation was performed to provide a more robust internal estimate of model performance and to reduce optimism from model selection. The repeated nested cross-validation framework achieved an AUC of 0.788 (95% CI: 0.710–0.856), accuracy of 0.750 (95% CI: 0.684–0.816), sensitivity of 0.684 (95% CI: 0.559–0.796), specificity of 0.789 (95% CI: 0.705–0.868), precision of 0.661 (95% CI: 0.532–0.781), and F1-score of 0.672 (95% CI: 0.566–0.760). The Brier score was 0.183 (95% CI: 0.158–0.210) (Table 5).

For threshold-dependent metrics, a prespecified default probability threshold of 0.50 was used. Predicted probabilities of 0.50 or greater were classified as complicated appendicitis, whereas probabilities below 0.50 were classified as uncomplicated appendicitis. AUC was calculated from continuous predicted probabilities and was not dependent on this threshold.

Logistic regression was the most frequently selected model across the 50 outer folds, being selected in 26 folds, followed by random forest in eight folds, XGBoost in seven folds, SVM in five folds, and gradient boosting in four folds (Table 6).

Calibration analysis showed a calibration intercept of −0.279 (95% CI: −0.677 to 0.098) and a calibration slope of 1.243 (95% CI: 0.825–1.809). The calibration plot demonstrated moderate agreement between predicted and observed risk, although deviation from perfect calibration was observed in some probability ranges. These findings indicate useful discrimination and acceptable internal calibration, but predicted probabilities should be interpreted cautiously until external validation is performed (Table 7).

Benchmark analysis was performed to contextualize the clinical usefulness of the complicated appendicitis prediction framework. A modified Pediatric Appendicitis Score (modified PAS) was retrospectively calculated using available dataset variables. The modified PAS achieved an AUC of 0.672 for complicated appendicitis prediction. A parsimonious conventional logistic regression model using symptom duration, fever documented in the emergency room, WBC count, NLR, CRP, and appendicolith by radiology achieved an AUC of 0.807, accuracy of 0.763, sensitivity of 0.702, specificity of 0.800, precision of 0.678, F1-score of 0.690, and Brier score of 0.178. However, because several original PAS components were unavailable or inconsistently documented and required assumptions, this result should be interpreted only as a limited exploratory comparison and not as an estimate of the performance of a prospectively measured PAS. The broader machine learning framework showed performance comparable to the parsimonious logistic regression model (Table 8).

**Figure 4:**
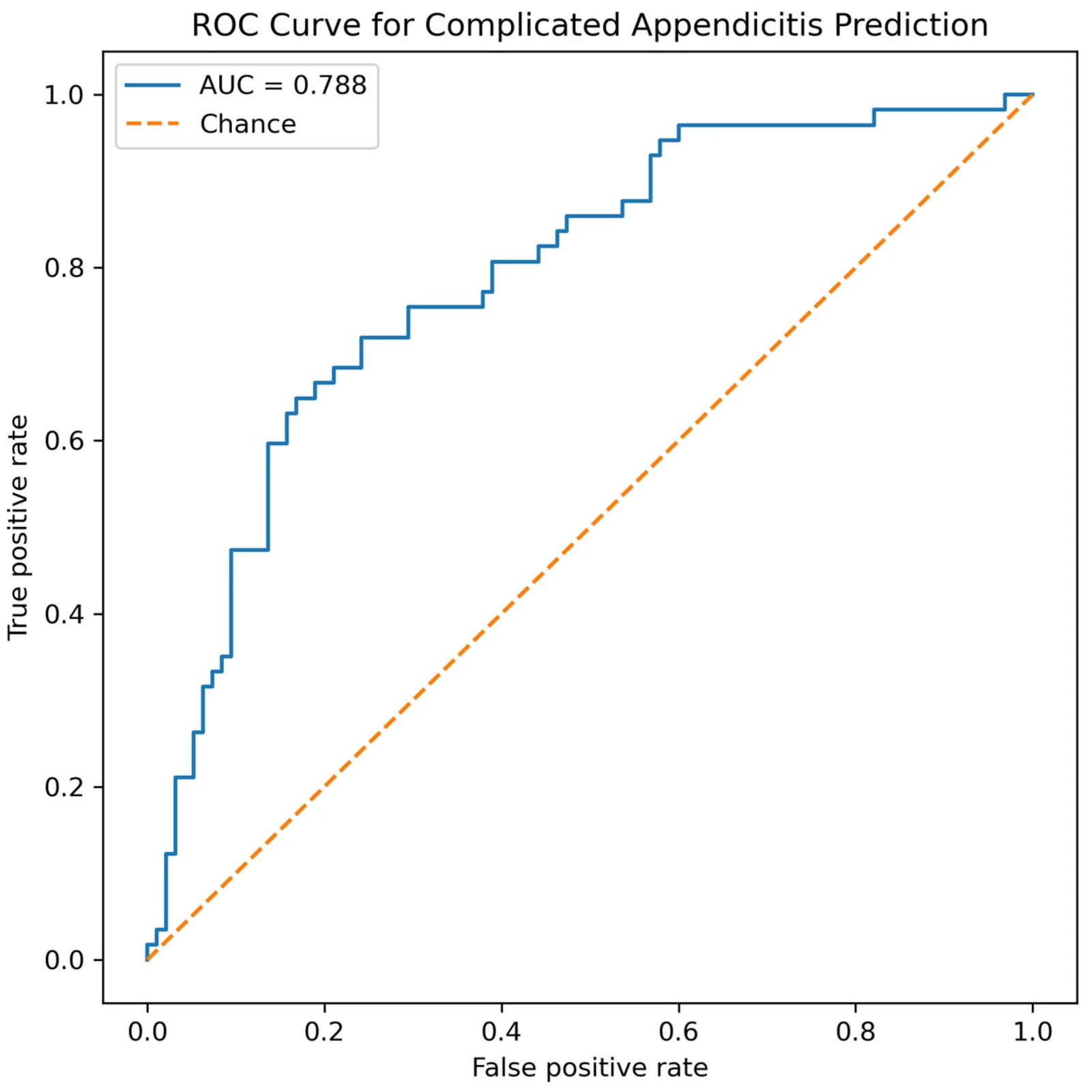
Receiver operating characteristic curve for complicated appendicitis prediction.

The receiver operating characteristic curve generated from patient-level out-of-fold predicted probabilities demonstrated an AUC of 0.788 for complicated appendicitis prediction (Figure 4).

**Figure 5:**
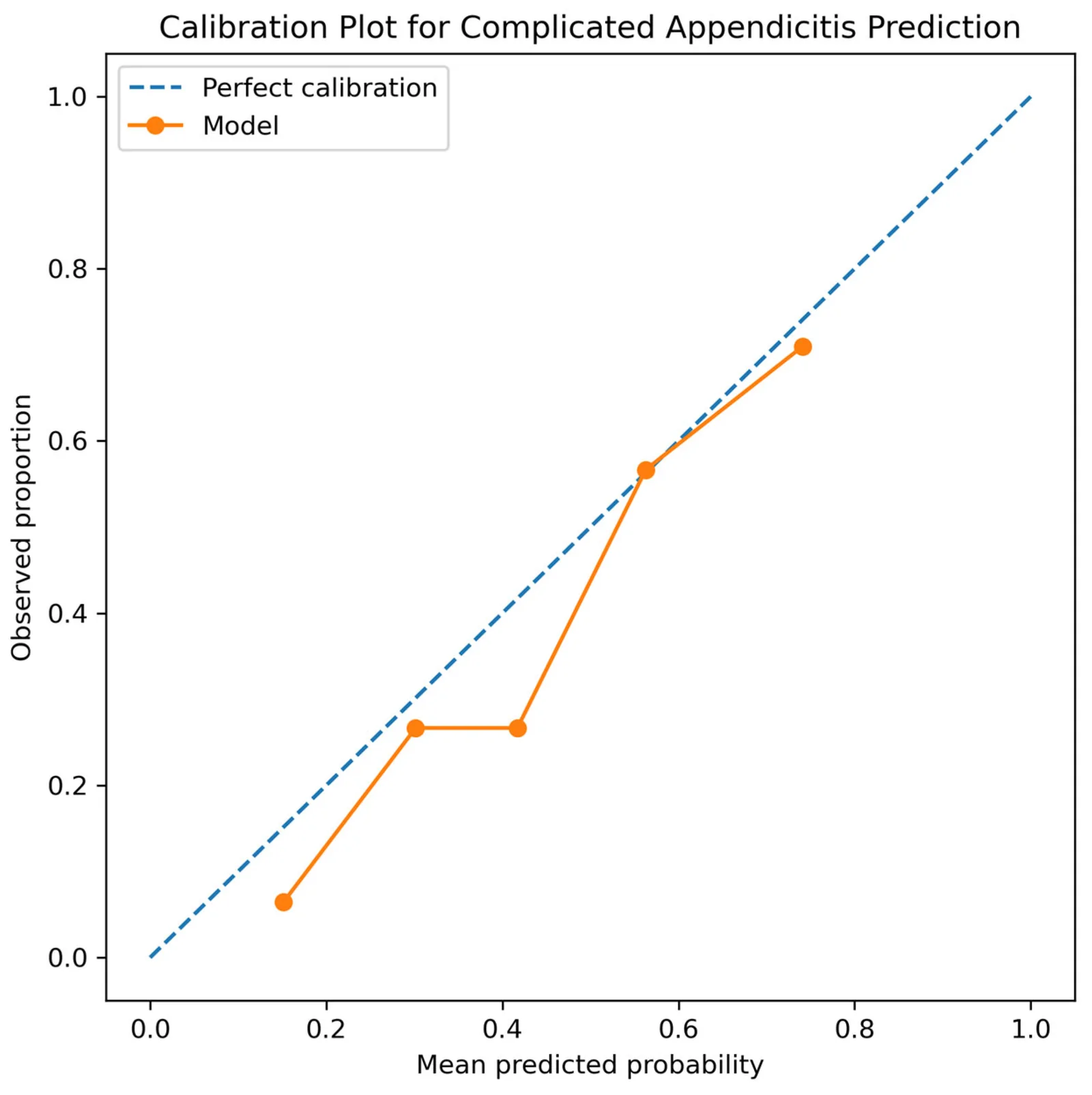
Calibration plot for complicated appendicitis prediction.

In Figure 5, predicted probabilities were generated using repeated nested cross-validation and averaged at the patient level. The dashed diagonal line represents perfect calibration. The Brier score was 0.183, calibration intercept was −0.279, and calibration slope was 1.243.

### 3.6. Machine Learning Performance for Expected LOS Prediction

Expected hospital length of stay (LOS) was modeled as a pre-treatment regression task using variables available after the initial clinical assessment, first laboratory investigations, and initial diagnostic imaging, but before surgery or definitive conservative treatment. Candidate predictors included age, sex, presenting symptoms, symptom duration, initial vital signs, first laboratory results, and pre-treatment radiological findings, including radiological perforation and intra-abdominal or pelvic collection when documented on initial imaging. Final complicated appendicitis status, operative findings, histopathological findings, postoperative variables, treatment-response variables, hospitalization-course variables, and actual LOS-related variables were excluded from the predictor set. Hospital LOS was used only as the continuous regression outcome and was not included as an input predictor.

Because radiological perforation and intra-abdominal or pelvic collection were included only when documented on initial diagnostic imaging, the LOS model should be interpreted as a pre-treatment post-imaging prediction model, not as a model available at the moment of admission before imaging.

Repeated nested cross-validation was performed to provide an internally validated estimate of expected LOS prediction performance and to reduce optimism from model selection. The repeated nested cross-validation regression framework achieved an MAE of 2.150 days (95% CI: 1.632–2.846), MdAE of 1.305 days, RMSE of 4.329 days (95% CI: 2.290–6.297), and R^2^ of 0.292 (95% CI: 0.213–0.481), as shown in Table 9.

To contextualize the added predictive value of the regression framework, performance was compared with two null baseline models. The first null model predicted the cohort median LOS of 3 days for all patients, while the second null model predicted the cohort mean LOS of 4.45 days for all patients. The machine learning framework achieved a lower MAE than both null baseline models, with an approximate 24.5% reduction in MAE compared with the median LOS baseline and a 33.4% reduction compared with the mean LOS baseline (Table 10). These findings indicate that the pre-treatment predictor set provided added predictive value beyond simple cohort-level LOS estimation.

Because LOS was right-skewed and included one extreme long-stay observation of 42 days, sensitivity analyses were performed. When the 42-day observation was excluded from performance evaluation, MAE decreased from 2.150 to 1.943 days, MdAE decreased from 1.305 to 1.282 days, RMSE decreased from 4.329 to 3.384 days, and R^2^ increased from 0.292 to 0.336. These findings indicate that the extreme 42-day observation contributed substantially to RMSE but had a smaller effect on MAE and MdAE.

A log-transformed LOS sensitivity model was also evaluated using $\log\left(LOS+1\right)$ as the regression outcome, with predictions back-transformed to days. The log-transformed model achieved an MAE of 2.072 days, MdAE of 1.123 days, RMSE of 4.377 days, and R^2^ of 0.276. Compared with the primary model, the log-transformed model slightly improved MAE and MdAE but did not improve RMSE or R^2^ (Table 11).

Sensitivity analyses were performed to assess the influence of LOS skewness and the extreme 42-day observation on model performance. The exclusion analysis showed that the 42-day observation increased overall prediction error, particularly RMSE. The log-transformed sensitivity model improved median error but did not improve overall variance explained.

Gradient boosting regression was the most frequently selected model across the 50 outer folds, being selected in 32 folds, followed by random forest regression in 18 folds. Ridge regression was not selected in any outer fold (Table 12).

These results indicate that pre-treatment predictors explained a modest-to-moderate proportion of LOS variability. Because LOS was right-skewed, with a small number of extreme long-stay observations, prediction was less accurate for patients with very prolonged hospitalization. Therefore, the LOS model should be interpreted as an early estimate of hospitalization burden rather than a precise discharge-date prediction model.

**Figure 6:**
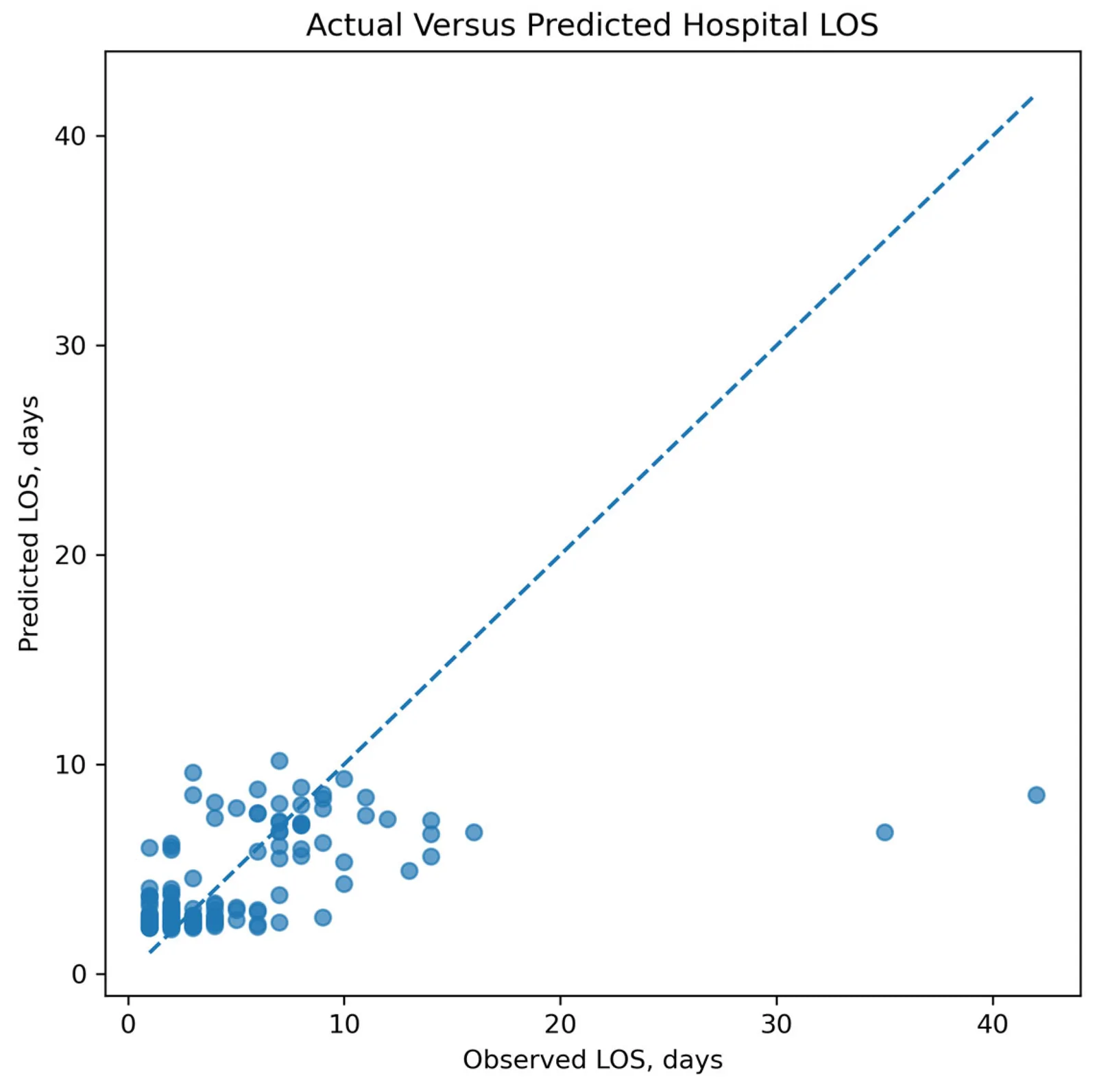
Actual versus predicted hospital length of stay.

The actual-versus-predicted LOS plot showed reasonable prediction for short-to-moderate hospital stays, but extreme long-stay observations were underpredicted, consistent with the right-skewed LOS distribution (Figure 6).

Predictions were generated using patient-level out-of-fold predictions from repeated nested cross-validation. The dashed diagonal line represents perfect prediction. The model showed reasonable estimation for short-to-moderate LOS values but underpredicted extreme long-stay observations.

### 3.7. Feature Importance and Explainable AI Results

Explainability analyses were performed for the two final prediction tasks: complicated appendicitis and expected hospital length of stay (LOS). For complicated appendicitis prediction, logistic regression was selected for explainability because it was the most frequently selected classification model across the repeated nested cross-validation outer folds and provides transparent coefficient-based interpretation. For expected LOS prediction, gradient boosting regression was selected for explainability because it was the most frequently selected regression model across the repeated nested cross-validation outer folds.

**Figure 7:**
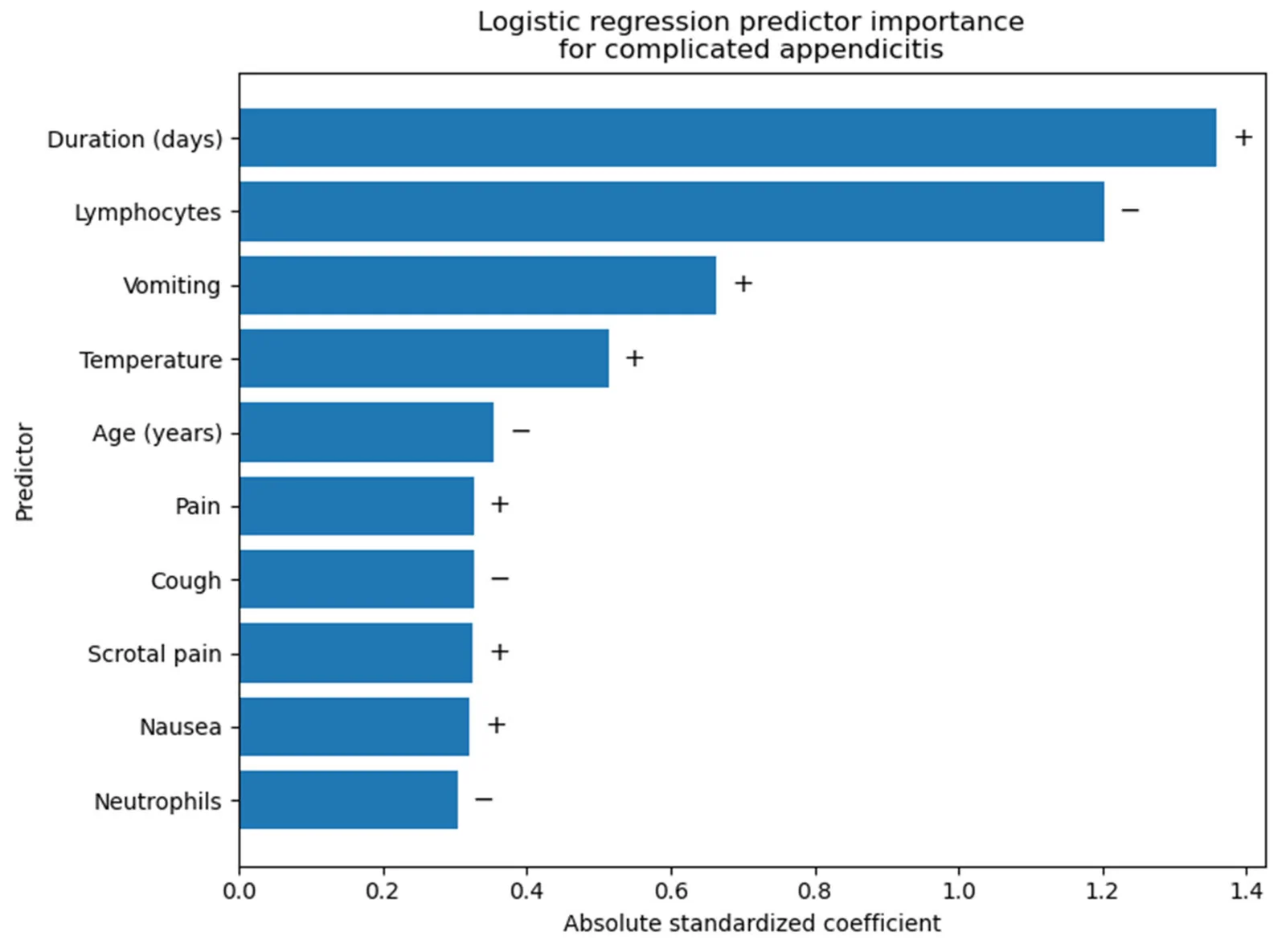
Logistic regression predictor importance for complicated appendicitis prediction.

For complicated appendicitis, standardized logistic regression coefficients identified symptom duration, lymphocyte percentage, vomiting, temperature, and age among the most influential predictors. Longer symptom duration, vomiting, and higher temperature increased the predicted log-odds of complicated appendicitis, whereas higher lymphocyte percentage and older age decreased the predicted log-odds (Figure 7).

Predictors are ranked according to the absolute magnitude of their standardized logistic regression coefficients. Larger values indicate greater contribution to the model. The plus (+) and minus (−) signs indicate whether higher predictor values increased or decreased the predicted log-odds of complicated appendicitis, respectively.

SHAP analysis for complicated appendicitis similarly identified symptom duration, lymphocyte percentage, vomiting, temperature, and age as important contributors to individual predictions. Positive SHAP values increased the predicted probability of complicated appendicitis, whereas negative SHAP values decreased the predicted probability.

**Figure 8:**
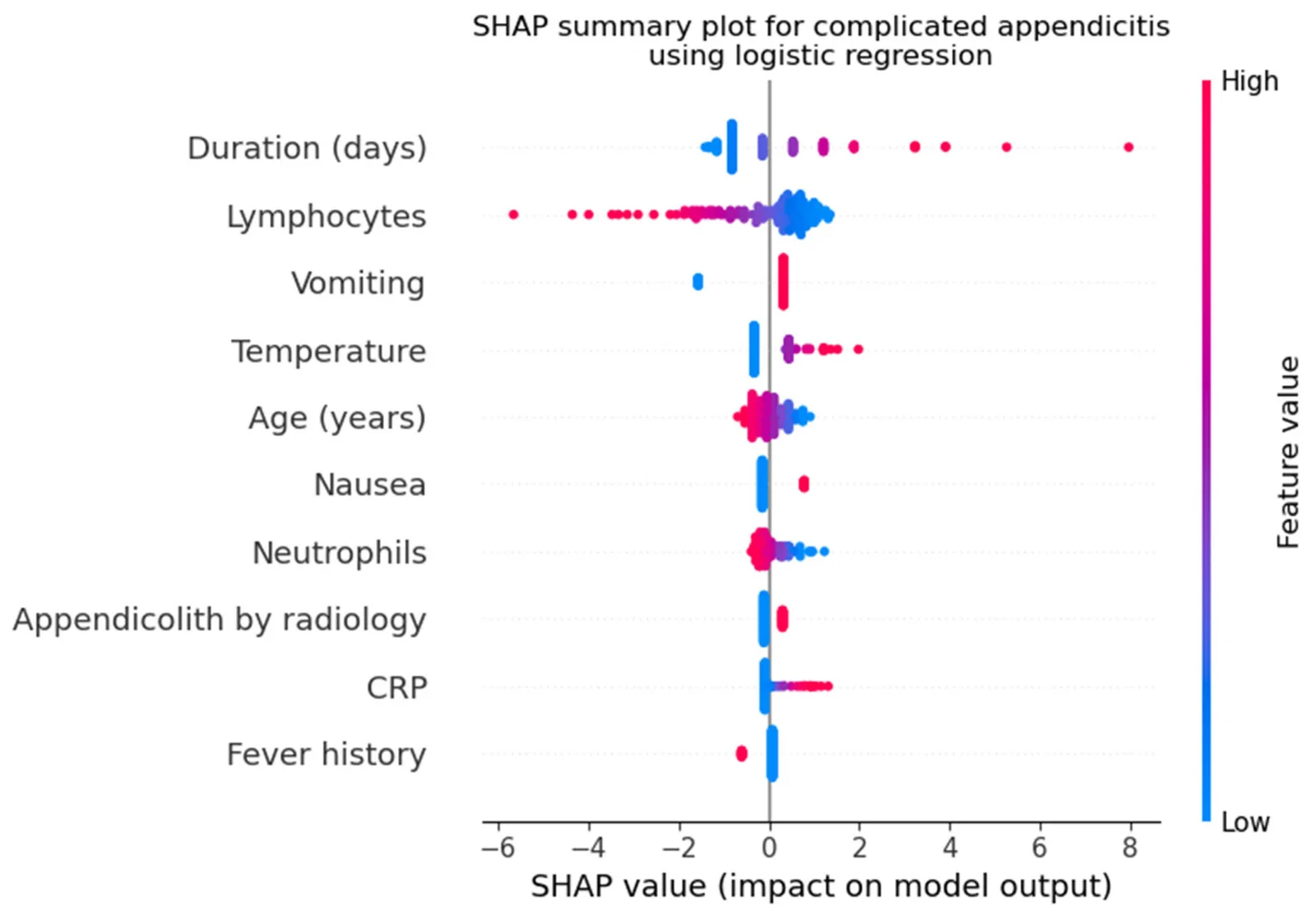
SHAP summary plot for complicated appendicitis prediction using logistic regression.

Each point represents one patient. Predictors are ordered according to their mean absolute SHAP values. Positive SHAP values increase the model output for complicated appendicitis, whereas negative values decrease it. Red indicates higher predictor values, and blue indicates lower values (Figure 8).

**Figure 9:**
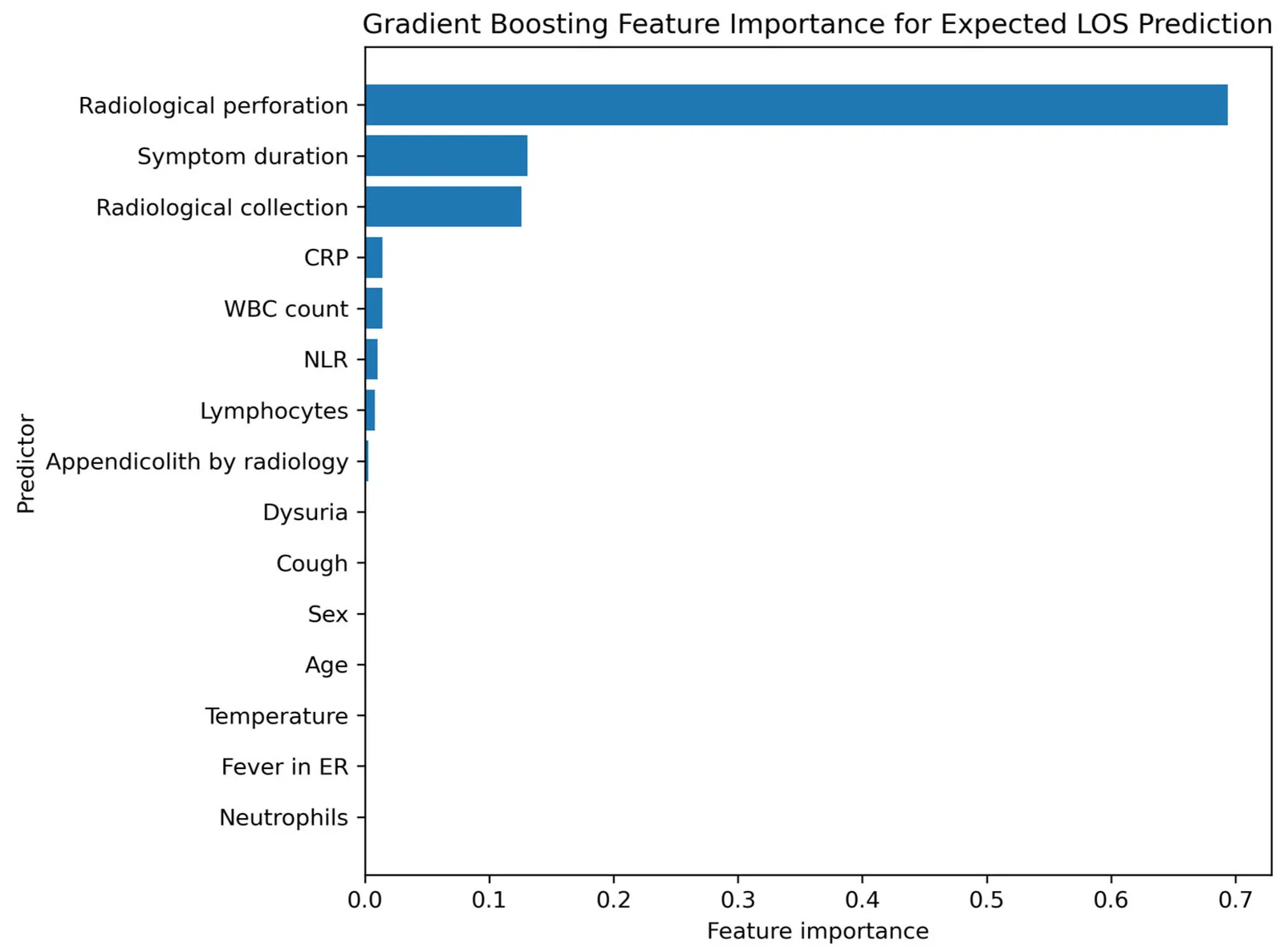
Gradient boosting regression feature importance for expected hospital LOS prediction.

For expected LOS prediction, gradient boosting feature importance identified pre-treatment radiological severity findings as the leading contributors to predicted LOS. Radiological perforation was the dominant predictor, followed by symptom duration and radiological collection. Inflammatory markers, including CRP, WBC count, NLR, and lymphocyte percentage, showed smaller contributions, while appendicolith by radiology had minimal contribution (Figure 9).

Predictors are ranked according to their relative contribution to the final gradient boosting regression model. Radiological perforation was the dominant predictor, followed by symptom duration and radiological collection. Feature-importance values indicate the magnitude of each predictor’s contribution but do not indicate whether the predictor increased or decreased predicted LOS.

**Figure 10:**
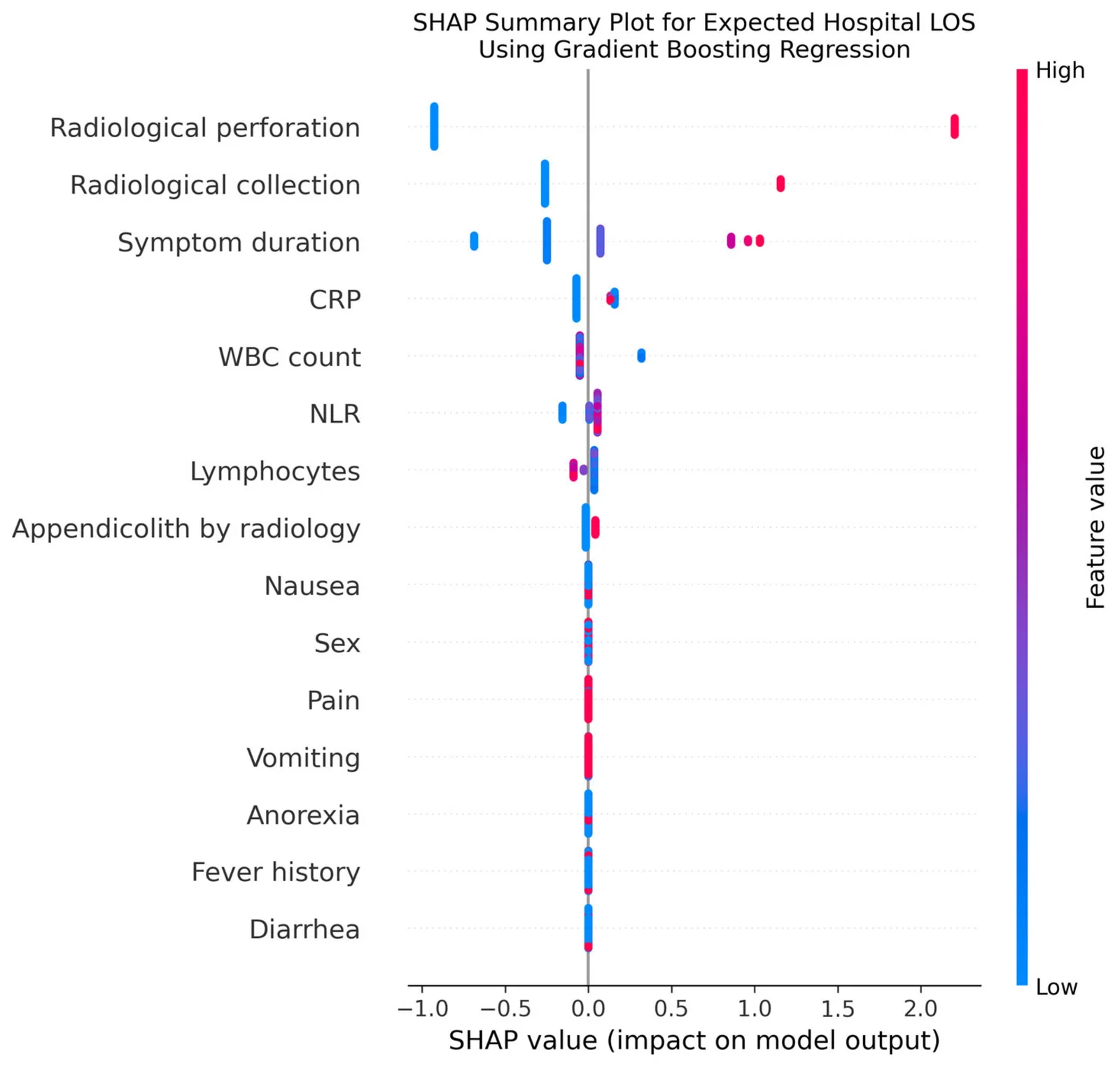
SHAP summary plot for expected hospital LOS prediction using gradient boosting regression.

SHAP analysis for expected LOS showed a similar pattern. Radiological perforation, radiological collection, and longer symptom duration were the strongest contributors toward longer predicted LOS. Higher inflammatory markers, including CRP, WBC count, NLR, and lymphocyte percentage, also contributed to predicted LOS but with smaller magnitude. Appendicolith by radiology showed minimal contribution (Figure 10).

Each point represents one patient. Positive SHAP values indicate contributions toward longer predicted LOS, whereas negative values indicate contributions toward shorter predicted LOS. Red indicates higher predictor values and blue indicates lower values. Radiological perforation, radiological collection, and symptom duration showed the largest contributions to predicted LOS (Table 13).

These findings suggest that the models captured clinically plausible predictive patterns involving delayed presentation, systemic inflammatory response, and anatomical disease severity documented before treatment. Differences between coefficient-based importance, tree-based feature importance, and SHAP rankings are expected because these methods quantify predictor contribution differently. Regression coefficients, feature-importance values, and SHAP values represent predictive associations within the fitted models and should not be interpreted as independent causal effects.

## 4. Discussion

This exploratory, retrospective, single-center study internally evaluated explainable machine learning methods for predicting complicated appendicitis and expected hospital length of stay (LOS) in children. Pre-treatment clinical and laboratory variables provided moderate discrimination for complicated appendicitis, whereas post-assessment variables incorporating selected radiological findings showed modest predictive value for LOS. The explainability analyses identified clinically plausible patterns involving symptom duration, inflammatory burden, and radiological disease severity. Importantly, the broader machine learning framework did not clearly outperform a parsimonious logistic regression model for complicated appendicitis, while the LOS framework improved prediction compared with simple cohort-level baselines.

Of 200 screened pediatric appendicitis records, 152 patients were included in the final analytical cohort after applying the predefined exclusion criteria. The cohort included children aged 4–14 years who were managed surgically or conservatively. The median symptom duration was 1 day, the median LOS was 3 days, and 57 patients (37.5%) were classified as having complicated appendicitis. These characteristics support the relevance of the cohort for exploring disease-severity and hospitalization-burden prediction, although its modest size limits the stability and generalizability of the developed models.

Patients with complicated appendicitis had longer symptom duration, higher WBC count, higher neutrophil percentage, lower lymphocyte percentage, higher NLR, and longer LOS than those with uncomplicated appendicitis. Fever documented in the emergency department was also more frequent in the complicated group. These findings are clinically plausible and support the relationship between delayed presentation, systemic inflammatory response, and progression to more severe appendiceal disease.

LOS was highly right-skewed, with most patients discharged within the first few days and a small number experiencing prolonged hospitalization. The median LOS was 7 days in patients with complicated appendicitis compared with 2 days in those with uncomplicated disease. This substantial difference confirms that LOS reflects not only the presence of appendicitis but also disease severity, treatment complexity, recovery, and resource requirements. Modeling LOS as a continuous outcome therefore provided a more informative representation of hospitalization burden than categorizing patients using a single prolonged-stay threshold.

A major methodological feature was the use of a single prespecified prediction time point after initial clinical assessment, first laboratory testing, and initial diagnostic imaging, but before surgery or definitive conservative treatment. Accordingly, the models were designed as exploratory post-assessment, pre-treatment prediction frameworks and, after further refinement and external validation, may support clinical decision-making at this stage rather than at initial admission.

Complicated appendicitis was defined using predefined radiological, operative, and histopathological criteria. For surgically managed patients, operative and histopathological findings were used to establish the outcome, whereas imaging-based severity findings were used for conservatively managed patients. Outcome-defining variables were not entered into the complicated appendicitis prediction model, thereby reducing target leakage and circular prediction.

The repeated nested cross-validation framework achieved an AUC of 0.788, accuracy of 0.750, sensitivity of 0.684, specificity of 0.789, and Brier score of 0.183. These results indicate moderate discrimination, with somewhat stronger performance for identifying uncomplicated cases than for detecting all complicated cases. The calibration intercept of −0.279 and calibration slope of 1.243 suggested moderate agreement between predicted and observed risk, although deviations from ideal calibration remained. The resulting patient-level probabilities should therefore be interpreted cautiously.

The incompletely reconstructed PAS achieved an AUC of 0.672. However, several original PAS components were unavailable or inconsistently documented and required assumptions during retrospective reconstruction. This result should therefore be regarded only as a limited contextual comparison and should not be interpreted as evidence that the machine learning framework outperformed a prospectively and completely measured PAS. Moreover, PAS was originally developed to estimate the probability of appendicitis rather than to distinguish complicated from uncomplicated disease.

More importantly, the parsimonious logistic regression model achieved an AUC of 0.807, accuracy of 0.763, sensitivity of 0.702, specificity of 0.800, and Brier score of 0.178. Its performance was comparable to, and slightly better than, that of the broader machine learning framework. Logistic regression was also the most frequently selected classification algorithm across the outer cross-validation folds. Together, these findings suggest that complex nonlinear methods did not provide clear incremental value over a simpler, clinically interpretable model in this modest-sized cohort. Variables such as symptom duration, fever in the emergency department, WBC count, NLR, CRP, and appendicolith may therefore provide useful risk-stratification information when modeled transparently.

For expected LOS prediction, the repeated nested cross-validation framework achieved an MAE of 2.150 days, MdAE of 1.305 days, RMSE of 4.329 days, and $R^{2}$ of 0.292. Performance was better for short-to-moderate hospital stays, whereas extreme long-stay observations were underpredicted. This is clinically understandable because prolonged hospitalization may depend on postoperative complications, treatment response, recovery trajectory, discharge barriers, and institutional factors that were intentionally excluded from the pre-treatment predictor set.

The LOS framework improved MAE by approximately 24.5% compared with the cohort median baseline and by 33.4% compared with the cohort mean baseline. These results indicate that post-assessment clinical, laboratory, and radiological information provided predictive value beyond assigning the same expected LOS to every patient. However, the modest $R^{2}$ demonstrates that a substantial proportion of LOS variability remained unexplained.

The findings highlight the potential of explainable machine learning models to provide an early estimate of expected hospital LOS after initial clinical assessment and imaging, which may eventually support bed allocation, resource planning, and family counseling.

However, this potential remains exploratory, and external validation and clinical utility assessment are required before the model can be incorporated into practice. The LOS estimates should therefore be viewed as an early indication of hospitalization burden rather than as precise predictions of discharge dates.

Sensitivity analyses clarified the influence of the extreme 42-day hospitalization. Excluding this observation reduced MAE from 2.150 to 1.943 days and RMSE from 4.329 to 3.384 days, while MdAE changed only slightly from 1.305 to 1.282 days. This indicates that the extreme case had a greater effect on squared-error measures than on median-based error.

The log-transformed LOS model reduced MAE to 2.072 days and MdAE to 1.123 days but did not improve RMSE or $R^{2}$. These findings suggest that the framework was relatively robust for typical hospital stays but remained limited in predicting rare, very prolonged admissions.

Explainability analyses supported the clinical plausibility of both prediction tasks. For complicated appendicitis, symptom duration, lymphocyte percentage, vomiting, temperature, and age were among the most influential predictors. Longer symptom duration, vomiting, and higher temperature were associated with increased predicted risk, whereas higher lymphocyte percentage was associated with lower risk.

For LOS prediction, radiological perforation was the dominant predictor, followed by symptom duration and radiological intra-abdominal or pelvic collection. CRP, WBC count, NLR, and lymphocyte percentage contributed with smaller magnitudes. These findings suggest that expected hospitalization burden was influenced by both anatomical severity and systemic inflammatory response. Nevertheless, standardized coefficients, feature-importance values, and SHAP values represent predictive associations within the fitted models and should not be interpreted as independent causal effects.

The study has several strengths. Cohort selection, study setting, inclusion criteria, and exclusion reasons were transparently reported. Predictor eligibility was aligned with a clearly defined clinical time point, and outcome-defining variables were excluded from the complicated appendicitis predictor set. LOS was analyzed continuously rather than duplicated as a binary prolonged-hospitalization endpoint. Repeated nested cross-validation separated hyperparameter selection from performance estimation, while bootstrap confidence intervals, calibration analysis, benchmark comparisons, sensitivity analyses, and explainability methods improved the transparency of model evaluation.

Several limitations should be acknowledged. This was an exploratory, retrospective, single-center study involving only 152 patients. Although repeated nested cross-validation reduced optimism associated with model and hyperparameter selection, it cannot eliminate overfitting or establish generalizability. The findings represent internal estimates of model behavior within the available cohort rather than definitive measures of real-world performance.

No independent external validation cohort was available. Consequently, the models have not been shown to perform consistently across hospitals, patient populations, laboratory systems, imaging practices, treatment protocols, or discharge pathways. This limitation is particularly important for LOS because hospitalization duration is strongly affected by local clinical and administrative practices. The models should therefore not be considered ready for clinical implementation.

All patients underwent ultrasound as the initial imaging modality, while CT was performed selectively when an intra-abdominal or pelvic collection was identified or suspected. This ultrasound-first pathway reflects routine clinical practice but introduces imaging heterogeneity because CT may characterize perforation and collection-related findings more accurately than ultrasound. Radiological variables were extracted from the original clinical reports without independent image rereview, which may have introduced reporting variability and limited reproducibility.

Formal patient-level prediction intervals were not estimated for LOS. Although MAE, MdAE, RMSE, $R^{2}$, and sensitivity analyses describe overall model performance, they do not communicate the uncertainty surrounding an individual patient’s predicted LOS. This uncertainty would be important if estimates were used for family counseling, bed management, or resource allocation.

Decision-curve analysis was also not performed. Therefore, the study did not evaluate threshold-specific net clinical benefit or demonstrate that use of the model would improve decisions compared with current practice. Future studies should incorporate prediction intervals or conformal-prediction approaches and evaluate decision curves before clinical integration is considered.

Future research should prospectively validate these findings in larger multicenter pediatric cohorts. External validation should assess discrimination, calibration, prediction error, subgroup performance, model stability, and clinical utility across different healthcare settings. Future stage-specific models could separately evaluate admission-only predictors, post-imaging variables, postoperative factors, and discharge-readiness indicators. Additional variables such as structured physical examination findings, complete PAS and pARC components, appendiceal diameter, abscess size, serial inflammatory markers, treatment response, oral intake, pain scores, and discharge barriers may improve prediction when collected at appropriate clinical time points.

In summary, routinely available post-assessment, pre-treatment variables showed potential for estimating complicated appendicitis risk and expected LOS. The broader machine learning framework did not clearly outperform parsimonious logistic regression for complicated appendicitis, but the LOS framework improved prediction beyond simple mean and median baselines. These results support continued investigation of transparent and stage-specific prediction approaches while emphasizing that prospective multicenter external validation and clinical utility assessment are required before implementation.

## 5. Conclusions

This exploratory, retrospective, single-center study internally evaluated explainable machine learning methods for predicting complicated appendicitis and expected hospital length of stay in children using routinely available clinical, laboratory, and radiological variables. The models identified clinically plausible patterns related to symptom duration, inflammatory burden, and radiological disease severity. For complicated appendicitis, the broader machine learning framework achieved moderate discrimination but did not clearly outperform the parsimonious logistic regression benchmark, indicating that a simpler and more interpretable model may provide comparable value in a modest-sized cohort. For LOS, the machine learning framework improved prediction compared with cohort mean and median baselines, although performance remained limited for rare, very prolonged hospitalizations.

These findings suggest that, after further refinement, explainable prediction models may help estimate hospitalization burden following initial clinical assessment, laboratory testing, and diagnostic imaging, with potential future applications in bed allocation, resource planning, and family counseling. However, the models were developed as post-assessment, pre-treatment frameworks and should not be considered admission-triage tools or precise discharge-date predictors.

The findings remain exploratory because the study involved a single-center cohort of 152 patients and relied on internal validation without an independent external cohort. Repeated nested cross-validation, calibration analysis, bootstrap confidence intervals, benchmark comparisons, sensitivity analyses, and explainability methods improved methodological transparency but cannot establish generalizability or clinical utility. Prospective multicenter external validation, patient-level uncertainty estimation, and clinical utility assessment are required before these models can be considered for clinical implementation.

## Figures and Tables

**Table 1:** Detailed reasons for exclusion of screened records from the final analytical cohort.

Exclusion Category	Detailed Reason	Number (n)
Missing key outcome data	Hospital LOS not documented	4
	No documentation of perforation, gangrene, abscess, pus, phlegmon, or intra-abdominal collection	8
	**Total missing key outcomes**	**12**
Incomplete essential medical records	Age missing	1
	Numerical body temperature at presentation missing	21
	**Total incomplete essential records**	**22**
Duplicate encounters	**Total repeated records for the same clinical encounter**	**7**
Chronic conditions affecting LOS	Malignancy	1
	Immunodeficiency	2
	Congenital heart disease	1
	Sickle cell disease	3
	**Total chronic-condition exclusions**	**7**

Note: Bold values represent the subtotal number of excluded participants within each exclusion category; the sum of these subtotals gives the overall number of excluded participants.

**Table 2:** Predictor availability and inclusion by prediction task.

Predictor	Complicated Appendicitis ML Framework ^1^	Parsimonious Logistic Regression	Expected LOS ML Framework ^2^
Age	Yes	No	Yes
Sex	Yes	No	Yes
Abdominal pain	Yes	No	Yes
Vomiting	Yes	No	Yes
Anorexia	Yes	No	Yes
Fever history	Yes	No	Yes
Diarrhea	Yes	No	Yes
Nausea	Yes	No	Yes
Constipation	Yes	No	Yes
Dysuria	Yes	No	Yes
Cough	Yes	No	Yes
Scrotal pain	Yes	No	Yes
Symptom duration	Yes	Yes	Yes
Body temperature	Yes	No	Yes
Fever documented in the emergency department	Yes	Yes	Yes
WBC count	Yes	Yes	Yes
Neutrophil percentage	Yes	No	Yes
Lymphocyte percentage	Yes	No	Yes
Neutrophil-to-lymphocyte ratio	Yes	Yes	Yes
C-reactive protein	Yes	Yes	Yes
Appendicolith on initial imaging	Yes	Yes	Yes
Radiological perforation	No	No	Yes
Radiological intra-abdominal or pelvic collection	No	No	Yes

^1^ The same predictor set was used for logistic regression, support vector machine, random forest, gradient boosting, and XGBoost within the broader complicated appendicitis framework. ^2^ The same predictor set was used for ridge regression, random forest regression, and gradient boosting regression within the expected LOS framework.

**Table 3:** Baseline demographic, clinical, and laboratory characteristics of the study cohort.

Variable	Overall Cohort, n = 152
Age, years	9.64 ± 2.22
Male sex	100 (65.8%)
Female sex	52 (34.2%)
Symptom duration, days, median (IQR)	1 (1–3)
Fever (documented in the emergency room)	55 (36.2%)
Vomiting	130 (85.5%)
Anorexia	26 (17.1%)
WBC count	14.74 ± 5.73
Neutrophil percentage	77.89 ± 10.64
Lymphocyte percentage	12.86 ± 8.62
NLR	8.91 ± 5.68
CRP	0.67 (0–6.05)
LOS, days, median (IQR)	3 (2–6)

Values are presented as mean ± standard deviation, median (interquartile range), or n (%), as appropriate. WBC: white blood cell; NLR: neutrophil-to-lymphocyte ratio; CRP: C-reactive protein; LOS: length of stay.

**Table 4:** Comparison between uncomplicated and complicated appendicitis groups.

Variable	Uncomplicated, n = 95	Complicated, n = 57	*p*-Value
Age, years	9.91 ± 1.99	9.19 ± 2.52	0.072
Symptom duration, days	1 (1–2)	3 (1–4)	<0.001
Fever in ER	25 (26.3%)	30 (52.6%)	0.002
WBC count, ×10^9^/L	13.39 ± 5.06	17.00 ± 6.11	<0.001
Neutrophils, %	75.81 ± 11.96	81.37 ± 6.77	<0.001
Lymphocytes, %	15.14 ± 9.75	9.08 ± 4.18	<0.001
NLR	7.57 ± 5.07	11.15 ± 5.97	<0.001
CRP, mg/dL	0.29 (0–2.9)	2 (0–17)	0.054
Appendicolith by radiology	21 (22.1%)	20 (35.1%)	0.119
Perforation	0 (0.0%)	45 (78.9%)	<0.001
Radiological collection	0 (0.0%)	28 (49.1%)	<0.001
Pathological abscess	0 (0.0%)	1 (1.8%)	0.375
LOS, days	2 (1–3)	7 (4–9)	<0.001

Values are presented as mean ± standard deviation, median (interquartile range), or n (%), as appropriate. ER: emergency room; WBC: white blood cell count; NLR: neutrophil-to-lymphocyte ratio; CRP: C-reactive protein; LOS: length of stay. Severity-defining variables were included in this descriptive comparison but were excluded from the complicated appendicitis prediction model to reduce target leakage.

**Table 5:** Pre-treatment variables associated with complicated appendicitis.

Predictor	Odds Ratio	95% CI	*p*-Value
Symptom duration > 1 day	5.35	2.62–10.96	<0.001
Fever in ER	3.11	1.56–6.22	0.002
WBC count > 14.0 × 10^9^/L	2.10	1.07–4.10	0.044
NLR > 8.2	2.66	1.35–5.25	0.007
CRP > 0.67	1.32	0.69–2.56	0.503
Appendicolith by radiology	1.90	0.92–3.95	0.119

Odds ratios were calculated using univariate 2 × 2 analysis. CI: confidence interval; ER: emergency room; WBC: white blood cell count; NLR: neutrophil-to-lymphocyte ratio; CRP: C-reactive protein. Variables directly contributing to the definition of complicated appendicitis were excluded from this predictor table to reduce circular interpretation.

**Table 6:** Repeated nested cross-validated performance for complicated appendicitis prediction.

Metric	Estimate	95% CI
Accuracy	0.750	0.684–0.816
Sensitivity	0.684	0.559–0.796
Specificity	0.789	0.705–0.868
Precision	0.661	0.532–0.781
F1-score	0.672	0.566–0.760
AUC	0.788	0.710–0.856
Brier score	0.183	0.158–0.210
Calibration intercept	−0.279	−0.677–0.098
Calibration slope	1.243	0.825–1.809

Values were calculated from patient-level out-of-fold predictions generated using repeated nested cross-validation. Ninety-five percent confidence intervals were estimated using patient-level bootstrap resampling. AUC: area under the receiver operating characteristic curve.

**Table 7:** Model selection frequency across repeated nested cross-validation outer folds for complicated appendicitis prediction.

Model	Number of Outer Folds Selected
Logistic regression	26
Random forest	8
XGBoost	7
SVM	5
Gradient boosting	4

Values represent the number of outer test folds in which each model was selected by the inner cross-validation procedure. The repeated nested cross-validation framework included 50 outer test folds.

**Table 8:** Benchmark comparison for complicated appendicitis prediction.

Model	AUC	Accuracy	Sensitivity	Specificity	Precision	F1-Score	Brier Score
Modified PAS	0.672	0.658	0.526	0.737	0.545	0.536	—
Repeated nested CV ML framework	0.788	0.750	0.684	0.789	0.661	0.672	0.183
Parsimonious logistic regression	0.807	0.763	0.702	0.800	0.678	0.690	0.178

PAS: Pediatric Appendicitis Score. The modified PAS was retrospectively calculated using available dataset variables and should not be interpreted as a complete reconstruction of the original PAS. CV ML: cross-validation machine-learning. Brier score was not calculated for modified PAS because it is a point-based score rather than a calibrated probability model.

**Table 9:** Repeated nested cross-validated performance for expected hospital LOS prediction.

Metric	Estimate	95% CI
MAE, days	2.150	1.632–2.846
MdAE, days	1.305	1.004–1.493
RMSE, days	4.329	2.290–6.297
R^2^	0.292	0.213–0.481

MAE, MdAE, RMSE, and R^2^ were calculated from patient-level out-of-fold predictions generated using repeated nested cross-validation. Ninety-five percent confidence intervals were estimated using patient-level bootstrap resampling with 2000 bootstrap samples for MAE, MdAE, RMSE, and R^2^. MAE, MdAE, and RMSE are expressed in days. LOS: length of stay.

**Table 10:** Regression performance and null baseline comparison for expected hospital LOS prediction.

Model	MAE, Days	MdAE, Days	RMSE, Days	R^2^
Repeated nested CV ML framework	2.150	1.305	4.329	0.292
Median LOS null model	2.849	2.000	5.345	−0.080
Mean LOS null model	3.226	2.454	5.144	0.000

The median LOS null model predicted 3 days for every patient, and the mean LOS null model predicted 4.45 days for every patient. MAE, MdAE, RMSE, and R^2^ were calculated using observed hospital LOS as the reference outcome. MAE, MdAE, and RMSE are expressed in days. LOS: length of stay.

**Table 11:** Sensitivity analysis for expected hospital LOS prediction.

Analysis	MAE, Days	MdAE, Days	RMSE, Days	R^2^
Primary repeated nested CV model	2.150	1.305	4.329	0.292
Excluding 42-day LOS observation	1.943	1.282	3.384	0.336
Log-transformed LOS model, back-transformed	2.072	1.123	4.377	0.276

**Table 12:** Model selection frequency across repeated nested cross-validation outer folds for expected LOS.

Model	Number of Outer Folds Selected
Gradient boosting regression	32
Random forest regression	18
Ridge regression	0

Values represent the number of outer test folds in which each model was selected by the inner cross-validation procedure. The repeated nested cross-validation framework included 50 outer test folds.

**Table 13:** Top predictors identified by explainable machine learning analysis.

Outcome	Final Model Used for Explainability	Top Predictors
Complicated appendicitis	Logistic regression	Symptom duration, lymphocyte percentage, vomiting, temperature, age
Expected hospital LOS	Gradient boosting regression	Radiological perforation, symptom duration, radiological collection, CRP, WBC count, NLR, lymphocyte percentage, appendicolith by radiology

## Data Availability

Data are unavailable due to privacy and ethical restrictions.
